# Student Experiential Activities: A Novel Strategy to Teach Neuroanatomy in Lectures

**DOI:** 10.7759/cureus.50789

**Published:** 2023-12-19

**Authors:** Suresh Narayanan, Priyadharshini N Adikesavan, Vimala ananthy

**Affiliations:** 1 Department of Anatomy, All India Institute of Medical Sciences, Madurai, Ramanathapuram, IND; 2 Department of Anatomy, Sri Manakula Vinayagar Medical College and Hospital, Puducherry, IND; 3 Department of Pharmacology, Mahatma Gandhi Medical College and Research Institute, Puducherry, IND

**Keywords:** experiential learning, student engagement, activity-based teaching, lectures, neuroanatomy

## Abstract

Introduction

Activity-based teaching is a widely used pedagogical tool for enhancing anatomy learning. However, involving the learner in experiential activities in lectures is an unexplored area in medical research. The present study aims to determine whether incorporating student experiential activities into lectures impacts student’s learning of neuroanatomy.

Materials and methods

We used a pre-test/post-test experimental study design to compare the learning outcome between the experiential activity based lecture and traditional lectures. We divided 150 students into control (75 students) and intervention groups (75 students). To assess the baseline knowledge on the subject, the students attended 20 clinical scenario-based multiple-choice questions two weeks before the lectures. Then, both groups attended two lecture sessions on the functional areas of the brain. In the control group, the students drew the boundaries of a particular cerebral area and labeled its function and clinical symptoms. In the intervention group, the learners participated in experiential activities while following the instructions. The lecturer used their response as a scaffold to explain the function and clinical correlation of a specific part of the cerebral cortex. The same test questions were given to the students one week after the final lecture session to evaluate their level of understanding. We analyzed the scores of 111 students (57 students in the control group and 54 students in the interventional group) who attended the two lectures and pre- and post-test sessions. Students also completed a validated 10-item feedback questionnaire regarding their perception of the teaching sessions.

Results

The mean score improvement for the control and intervention groups was 4.86 ± 1.53 and 6.39 ± 2.93, respectively. The score improvement of the activity group was significantly higher than that of the control group (p = 0.006; d = 0.65). The perception scores of interest, knowledge attainment, and satisfaction were significantly higher in the intervention group compared to the control group.

Conclusion

The findings of this study suggest that experiential activities facilitate better comprehension of abstract neuroanatomical concepts as compared to traditional didactic teaching.

## Introduction

In recent years, there has been an increasing trend of neurological disorders in low- and middle-income countries [1]. To recognize the varied neurological symptoms and localize the brain damage, a physician must have a thorough understanding of the structural and functional aspects of the cerebral cortex [2]. However, medical students often view neuroanatomy as the most challenging part of the anatomy curriculum [3,4]. This neurophobia could be due to the perceived complexity of the topic, complex spatial orientation, and poor teaching methodologies [3,4]. Another concern is that educators and students view anatomy as a subject that needs memorization, which negatively influences both conceptual understanding and professional identity development in medical students. However, when anatomy content is delivered using novel, student-centered, integrated approaches, it enhances learning [5].

In the anatomy curriculum, lectures followed by dissection or prosection of specimens have been the standard method of teaching [5]. The lectures provide an opportunity to introduce the terminologies, test students’ prior knowledge, and discuss abstract concepts [6]. However, passivity in delivery, content overload, and lack of student participation are common factors that hinder the learning outcome [7]. Strategies such as case-based and team-based learning enable learners to generate plausible hypotheses or justify the reasoning behind the neurological symptoms for a better understanding of the subject content in a clinical context [8]. However, educators often witness students lacking interest and reduced participation during lecture discussions. This reluctance to participate in class discussions could be due to a lack of interest in the subject, the inherent difficulty in understanding the clinical correlation, and the depersonalized teaching nature [9,10]. Another concern for medical educators is to find a solution for emotionally engaging the millennial learners and provide them with a clear expectation of the neuroanatomy course [11]. Hence, it is imperative to develop educational strategies that create a relaxing learning environment, fostering clinical correlation of the subject content and addressing the students with varied learning skills [12-14].

Classroom demonstration refers to the activities designed by the instructor to promote the active participation of the learner and better conceptual understanding [15,16]. While some studies have shown that demonstrations can enhance student interest and explain concepts clearly [15-17], there is also evidence that class demonstrations can be ineffective [18]. However, in the medical curriculum, very few studies have explored the use of student-involved activities and their influence on the learning outcome [19,20]. Previous studies have focused on experiential learning activities such as wine tasting to sensitize the learner about the underlying neurosensory pathway and have used teacher-led demonstrations to provide functional and clinical relevance, creating a desire to learn [12,21]. Though these studies have reported that the activities enhanced the student interest in the subject, they have not addressed the efficacy of such experiential learning activities. Another study used gesture-based activities such as making funny faces, crying, and spitting saliva to symbolize the function of cranial nerves. The authors demonstrated that gestures and body movements helped the learners to acquire anatomical knowledge [20]. The activities presented in these articles are familiar to the learner, and the instructor needs to build new concepts around the activities. However, first-year students are relatively unfamiliar with the structure, function, and clinical presentation of individual areas of brain damage. Teaching the functional correlation of the brain and its clinical application in lectures is difficult because of delivering too much subject content in a limited amount of time (cognitive overload) and the abstract nature of the concept [4]. Hence, there is a need to design student participatory activities that emphasize the functional importance of the brain and its clinical relevance, thereby providing a holistic approach to learning.

The present study is designed on the self-determination theory of motivation. The self-determination theory states that providing a meaningful explanation of the subject content and creating an engaging learning environment enhance the motivational level of the learner [22]. This study aims to determine whether incorporating strategies such as student participatory activities into lectures impacts the student neuroanatomy learning experience. This intervention attempted to promote student interest in learning and simplify neuroanatomical concepts to alleviate neurophobia. The outcome of this study could be beneficial for medical students in easing their transition to clinical application and for educators in understanding the student engagement pattern during activity-based teaching in a lecture setting. The study objectives were to compare the test scores among students exposed to class activities with conventional teaching and To assess the perception of neuroanatomy teaching among students exposed to class activities with conventional teaching using a validated feedback questionnaire.

## Materials and methods

The present study was a pre-test/post-test experimental study conducted over eight weeks from September to October 2021. Before commencing the study, we obtained institutional research and ethical committee approval (EC-198/2021). The mean and standard deviation were derived from the pilot study, resulting in a sample size of 53 per group using the OpenEpi software. The study participants included first-year medical undergraduate students from a private medical college in Puducherry, India. The investigators explained the study protocol to the participants and obtained written consent. The investigators included first-year medical undergraduates who demonstrated a willingness to participate. All students joined medical training after completing their higher secondary education. The study participants had no prior exposure to the structure and functions of the cerebral cortex.

The authors reviewed anatomical books and research articles to standardize the neuroanatomical activities. We designed the activities based on the following criteria: a two-minute time limit, feasibility in the classroom, and the requirement of fewer resources (Table 1). Next, we framed 20 clinical scenario-based multiple-choice questions (MCQs) with five options, designed to minimize guessing. The stem of the MCQ consisted of a clinical case scenario, and the options included plausible cortical areas/gyri. The authors ensured that the MCQ assessed the learner's ability to connect the clinical symptoms with the damage to the functional area of the brain. Two senior anatomists reviewed the MCQs based on their relevance to the course material, difficulty level, and relationship to the activities. We chose MCQs as the mode of assessment because of their feasibility and objectivity in evaluating a large group of students. We developed the perception questionnaire through focus group discussions and checked for internal consistency using Cronbach's alpha (α = 0.880) with feedback from 15 students. The questionnaire used a 5-point Likert scale to measure students' perceptions regarding three aspects of teaching: 1) interest, 2) knowledge acquisition, and 3) satisfaction.

**Table 1:** Summary of class activities used for the intervention group

Cerebral area	Student activity
Orbitofrontal cortex	The Stroop test examines the ability of the patient to inhibit responses. We projected words such as green, red, and blue in different color fonts. Then, we asked the students to read the words, followed by the color of the word.
Lateral frontal cortex	The lecturer instructed the students to spell the word (WORLD) backward and recite the months of the year in reverse order.
Frontal eye field	The lecturer instructed the students to follow the movement of his finger from left to right.
Premotor area	We instructed the students to rapidly oppose their thumb to their index, middle, ring, and little fingers.
Inferior parietal lobule	1. The demonstration of object use is aimed at assessing ideational apraxia. We asked the students to demonstrate the usage of a pen or a toothbrush. 2. Imitation of gestures is used to evaluate ideomotor apraxia. We asked the students to replicate gestures, such as blowing out a candle or waving goodbye.
Right parietal lobe	The lecturer instructed the students to draw a clock from their memory.
Primary motor and sensory area	During the exercise, we asked the student volunteer to shut his eyes and stretch his arms out with his palms facing upward. Then, the instructor touched one of his fingers on his left hand and requested him to move the corresponding finger on his right hand.
Angular gyrus	We instructed the learners to calculate this problem with their eyes closed (to minimize their interaction with their peers and focus on the mathematical solving) - Think of a number between 0 to 9 - Add 7 to it - Multiply the answer by 9 - Add the individual numbers of the answer (if it is double or triple digits, add the individual numbers once again) - Your answer is 9!
Auditory association area	We instructed the students to close their eyes and listen to the audio clip. The instructor played the “Nokia” ringtone and asked the learners to name the ringtone and the quote associated with it.
Motor and sensory speech area	We instructed the students to observe a picture for 30 seconds. After that, we randomly selected a few students and asked them to describe the observed visuals. Later, two trained volunteers demonstrated the clinical presentation of motor and sensory aphasia. Other students commented on the speech activity.
Fusiform gyrus	We projected six Indian celebrities in a single slide. We instructed the learners to name the celebrities and the reason for their popularity.
Visual construction area	Ghent’s overlapping figure test to check for apperceptive visual agnosia. We asked the students to name all the objects in the drawing.
Visual association area	Columbia Mental Maturity Test to check for associative visual agnosia. We asked the students to identify the odd object.

We administered the pre-test questions two weeks before the lectures to assess the students' baseline knowledge. We selected the topic "Functional Areas of the Cerebrum" as it was appropriate for incorporating student-led activities into the classroom. The research team ensured uniformity of learning objectives, PowerPoint slides, and clinical case scenarios for both groups. The students were assigned to control and intervention groups based on their roll numbers. Both groups attended two 60-minute lecture sessions on consecutive days (taken by the first and second authors). For the control group, the instructor guided the students to outline the brain, mark the boundaries of the particular gyri, and label its functions and clinical symptoms. For example, as the instructor was explaining about the angular gyri, the students colored the angular gyri and annotated its role in "arithmetic calculation". The students then annotated "inability to do arithmetic calculation in mind" if the angular gyri was damaged. In the intervention group, the instructor assigned tasks for the activity group to perform. The lecturer used the students’ response as a scaffold to connect the functions and clinical relevance of the cerebral cortex. For example, the instructor presented a mathematical problem as a stimulus and explained the role of the angular gyri in mental calculation. Both groups discussed clinical case scenarios at the end of the lecture. The clinical case scenarios and the amount of time spent on the subject content were the same for both groups. The same test questions and perception questionnaire were given to the students one week after they had completed the last lecture. An external faculty member, blinded to the study objectives, collected the data. The control group was later exposed to the activities in a laboratory session to address ethical concerns.

To calculate the score improvement of a student, we used the formula: post-test score minus pre-test score. We then determined the mean and standard deviations for both the score improvement and perception scores. We used the Shapiro-Wilk test to assess the normality of the data, and the results showed that the distribution was non-normal. Therefore, we used the Mann-Whitney test to compare the difference in score improvement and perception scores between the two groups. As a measure of effect size, we calculated Cohen's d using the formula: d = (X1 - X2) / SDp, where X1 - X2 represents the difference between the group means, and SDp is the pooled standard deviation between groups. A research study suggests that an effect size of 0.2 is small, 0.5 is medium, and 0.8 is large [23]. For our statistical analysis, we used SPSS Version 23 (IBM Corp., Armonk, NY, USA). We considered a p-value of less than 0.05 to be statistically significant.

## Results

The age range of the study participants was 18-21 years. The learning outcome of 111 students who have attended two lectures and pre- and post-test sessions was analyzed (57 in the control group and 54 in the intervention group). Among them, 43 (38.7%) were male and 68 (61.3%) were female. The mean pre-test and post-test scores of the control group were 2.95 ± 1.71 and 7.81 ± 1.61, respectively. Likewise, the mean pre-test and post-test scores of the interventional group were 2.94 ± 1.61 and 9.33 ± 2.95, respectively. The mean score improvement for the control and interventional groups was 4.86 ± 1.53 and 6.39 ± 2.93, respectively. The score improvement was significantly higher in the activity group as compared to the didactic group (p = 0.006; d = 0.65) (Table 2).

**Table 2:** Comparison of score improvement between the control and intervention groups. *A p-value of <0.05 indicates a significant difference SD, standard deviation

Assessment	Lecture group (n=57), mean ± SD	Lecture with activity group (n=54), mean ± SD	Statistics Mann-Whitney test
Score improvement	4.86 ± 1.53	6.39 ± 2.93	p=0.006*

**Figure 1:**
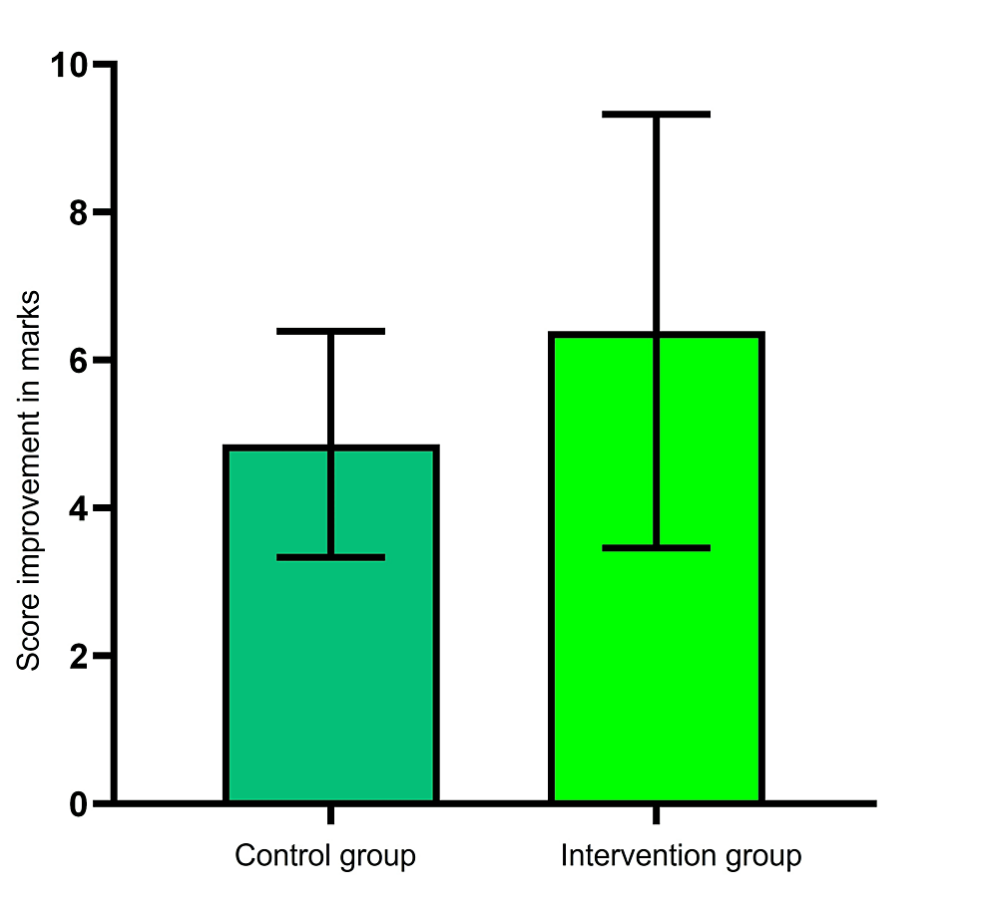
Graphical representation of score improvement between the control and intervention groups p=0.006

Figure 1 depicts the comparison of score improvement between the control group and the intervention group.

A total of 138 students attended at least one lecture, and among them, 125 students filled out the feedback questionnaire (66 students in the control group and 59 in the interventional group). The mean perception score of students in the intervention group was higher than 4. The perception score of the activity group was significantly higher for the ratings of interest, knowledge attainment, and satisfaction compared to the didactic group. However, we found no significant difference regarding the role of teaching sessions in clarifying the students' misconceptions of the subject content (Table 3).

**Table 3:** Comparison of perception scores between the control and intervention groups. *A p-value of <0.05 indicates a significant difference SD, standard deviation

Feedback statement	Lecture group (n=66), mean ± SD	Lecture with activity group (n=59), mean ± SD	Statistics
Enjoyability of the session	3.72 ± 0.9	4.62 ± 0.7	p<0.001*
Improvement of interest in neuroanatomy content	3.95 ± 1.1	4.38 ± 0.8	p=0.034*
Motivation to take part in neuroanatomy discussion	3.81 ± 1.0	4.18 ± 0.9	p=0.018*
Interactive learning environment	3.46 ± 1.0	4.50 ± 0.9	p<0.001*
Better understanding of functional areas of the brain than traditional lectures	3.92 ± 0.9	4.42 ± 0.8	p=0.001*
Improving my skills in localizing cerebral damage when compared to traditional lectures	3.60 ± 1.0	4.30 ± 0.7	p<0.001*
Clarifying my misconceptions about neuroanatomical concepts	3.78 ± 0.9	3.98 ± 0.9	p=0.211
Positive impact on my neuroanatomy learning experience	4.03 ± 0.9	4.42 ± 0.7	p=0.014*
Satisfied with this approach of neuroanatomy learning	3.80 ± 1.0	4.30 ± 0.8	p=0.002*
Similar method of teaching in other disciplines	3.83 ± 1.0	4.59 ± 0.8	p<0.001*

## Discussion

Our results have demonstrated that activity-based teaching leads to a moderate improvement in learning outcomes and enhances perceived interest, knowledge attainment, and satisfaction compared to conventional teaching. Similar findings were reported by Catena and Carbonneau, where the incorporation of hands-on activities such as electromyography during biomechanics, resulted in improved test scores [19]. Dao et al. used visually capturing scenery and wine tasting to elucidate and explain the neurosensory pathway for first-year medical students [12]. Similarly, Lewis et al. used teacher-led demonstrations to explain hematoma formation and the protective functions of cerebrospinal fluid to first- and fourth-year medical students in a large group setting [21]. The present study differs from the above-mentioned studies in both methodology and mode of assessment. In our teaching, the teacher gives instructions, and the learner attempts to accomplish the short in-class activities (2-3 minutes) that mimic real-world implications. Later, using their response as a scaffold, the lecturer correlated the function of a specific part of the cerebral cortex. Additionally, we have evaluated the learner's application of knowledge by using clinical case scenarios to offer an authentic learning experience.

The significantly higher knowledge gain in the intervention group could be due to the following factors:

1) Experiential learning: for instance, when discussing the prefrontal area, the students in the control group mapped the boundaries and labeled its function (decision-making, abstract thinking, and insight) and clinical relevance (socially inappropriate behavior). This method limits the learner's understanding and promotes rote learning because these statements resemble factual information. However, when students engage in an activity that requires them to make decisions, they can connect the action performed with the abstract concept [19]. These classroom activities likely address Kolb's learning cycle's experience (performing the task), reflection (reflecting on the task experience), conceptualization (linking activity and the concept), and experimentation components (applying in a clinical scenario) [17,19,24].

2) Increased motivation: Dickson and Stephens introduced deictic gestures (e.g., tapping the forehead, upper jaw, and lower jaw to indicate branches of the trigeminal nerve), representative gestures (e.g., placing a hand behind the ears to represent hearing), and metaphoric gestures (e.g., hands covering the eyes to simulate pupillary constriction) to mimic the functions of cranial nerves. The authors reported that students found the miming activity to be more interactive, engaging, and motivating than the didactic lecture [20]. As per self-determination theory, the instructional material needs to address the learner’s relatedness, autonomy, and competence to achieve motivation [22]. In the present study, performing the activities most likely created a sense of connectedness between the learner and the subject content/teacher. This could have heightened the interest and motivation to listen, thereby promoting better information encoding and conceptual understanding.

3) Student engagement: when students perform the activity, it breaks the traditional boundaries of the lecture setting and creates an exciting, curious, and collegial (performing the activities as a group) learning environment [15,21]. According to researchers, student engagement has three distinct dimensions, namely, behavioral (asking questions during a session), cognitive (willingness to exert the effort necessary to understand the subject), and emotional (interest and anxiety) [25,26]. The three phases of the activity, namely, the lecturer describing the task, and the learner performing, followed by focusing on the active part of the brain, reflect the emotional, behavioral, and cognitive components of engagement. Thus, these class activities were likely better at tapping into the three dimensions of student engagement than the didactic sessions [27].

4) Matching the learning style: each student has a preferred mode of instructional design that facilitates information acquisition. The activities described in the study create a unique multisensorial experience by activating the visual, auditory, and touch sensations, thereby matching their learning style [13,14,27]. The activities included varied tasks such as imitating a movement (kinesthetic), listening to ringtones (auditory), observing a picture (visual), and stating the color of the written words (reading). Since, a majority of learners prefer multimodal instructions [28], matching the learning style with instructional design could have resulted in the better acquisition of cognitive knowledge [24].

Research has stated that educators need to improve the student's state of mind in the classroom, maintain their interest, understand the ability of the learner to assimilate the knowledge, and encourage the real-world application of the subject content for effective student engagement [29]. Students in the activity group perceived that the lectures were more interesting, improved their knowledge acquisition, and provided higher satisfaction than the didactic group. The interest pattern, satisfaction, and perceived knowledge acquisition are indirect measures of student engagement and indicate that the hands-on activities heightened student engagement in the classroom. This higher rating could be due to the relaxed learning environment, the continued evoking of learners' interests, and the hands-on experience of the real-world application inculcated by the class activities. These findings are similar to other studies that reported positive student perceptions about using class demonstrations and their beneficial role in promoting learning [19,20]. However, there was no significant difference in the perceived ability of the teaching sessions to clarify the students' misconceptions. This finding suggests that students do not see the demonstration activities as a tool to clarify misconceptions but rather as a means of provoking interest or providing a contextual explanation.

The authors would like to point out that not all activities will have an equal impact on the student's learning experiences. During the lectures, the learners were much more interested in participating in the "Stroop test" and the "calculation task" than the "following the finger task." Therefore, certain activities created the desired excitement for the millennial learners, promoting participation out of genuine interest. This finding indicates that the nature of the task plays a crucial role in achieving student engagement. Therefore, educators need to design the activities based on the student's knowledge level, time availability, and the desired level of clinical correlation to achieve effective interaction. Lecturers should avoid excessive time spent on these activities as it could disrupt the delivery of the subject content.

The strategies presented in this article can serve as a practical guide for lecturers to develop and implement activity-based teaching in routine neuroanatomy teaching. Implementing participatory activities in lectures is easy and does not require extra time duration or additional faculty requirements. These activities serve as an effective pedagogical tool to teach clinical reasoning skills to novice learners, which enables them to correctly localize cerebral damage. Additional advantages include providing the learner with an enjoyable learning environment and creating a positive attitude towards the subject, thereby reducing the apprehensive nature of the learner and alleviating neurophobia. Aiding the learner in appreciating the relevance of neuroanatomy in these experiential activities, followed by clinical case discussion, can impart a collaborative and contextual learning experience [20,21]. Hence, the authors recommend that educators design class activities that can create an emotional connection with the learner, thereby creating cognitive engagement.

In our study, we measured the knowledge level after seven days because the Ebbinghaus curve indicates that most of the forgetting occurs during the first week after the learning process [30]. The study strengths include the large sample size, equal time allocation for the groups, and the measurement of application of the learned knowledge rather than recall of factual knowledge. The score improvement used in the study is a better indicator to assess the knowledge gained than the post-test scores, which might not reflect actual knowledge attainment.

The limitations include participants from a single institution, the limited number of teaching sessions (the authors were able to frame only three activities for basal ganglia and cerebellum lectures, and comparing the learning outcome between sessions with varied nonequivalent activities cannot give a valid result), and the interference of factors outside the study design. Though we tried to mimic the clinical presentation following cerebral damage, it cannot be equated to a case-based learning approach with discussion of clinical symptoms and radiological findings. Medical educators should consider these limitations before generalizing the findings for a broader interpretation.

The authors did not address long-term knowledge retention because the neuroanatomy module was the last module of the first-year curriculum, after which the study participants attended their university examination and moved to second-year teaching sessions (collecting post-test during the second year was not feasible). The teaching and assessment of the research project assumed that cerebral damage is limited to a specific brain area with no overlapping structures, which rarely occurs in clinical practice [2]. Future research can explore the replicability of the activities across various disciplines of basic science subjects, giving opportunities for students to create their activities and analyzing the impact of class demonstration types (observation, prediction, and experimentation) on knowledge retention.

## Conclusions

Our study has highlighted that student activities are an effective and engaging pedagogical tool to teach functional correlation of the cerebrum in lectures. This teaching method piqued the student's desire to learn neuroanatomy and improved the learning outcome in a classroom setting. Hence, carefully planned student activities can promote active participation, better understanding, and application of the subject content in a time-constrained curriculum.
